# Real-Time Dynamic Intelligent Image Recognition and Tracking System for Rockfall Disasters

**DOI:** 10.3390/jimaging10040078

**Published:** 2024-03-26

**Authors:** Yu-Wei Lin, Chu-Fu Chiu, Li-Hsien Chen, Chao-Ching Ho

**Affiliations:** 1 Department of Mechanical Engineering, National Taipei University of Technology, Taipei City 10608, Taiwan; 2 Department of Civil Engineering, National Taipei University of Technology, Taipei City 106344, Taiwan

**Keywords:** deep learning, rock detection, rock tracking, geology, machine vision

## Abstract

Taiwan, frequently affected by extreme weather causing phenomena such as earthquakes and typhoons, faces a high incidence of rockfall disasters due to its largely mountainous terrain. These disasters have led to numerous casualties, government compensation cases, and significant transportation safety impacts. According to the National Science and Technology Center for Disaster Reduction records from 2010 to 2022, 421 out of 866 soil and rock disasters occurred in eastern Taiwan, causing traffic disruptions due to rockfalls. Since traditional sensors of disaster detectors only record changes after a rockfall, there is no system in place to detect rockfalls as they occur. To combat this, a rockfall detection and tracking system using deep learning and image processing technology was developed. This system includes a real-time image tracking and recognition system that integrates YOLO and image processing technology. It was trained on a self-collected dataset of 2490 high-resolution RGB images. The system’s performance was evaluated on 30 videos featuring various rockfall scenarios. It achieved a mean Average Precision (mAP50) of 0.845 and mAP50-95 of 0.41, with a processing time of 125 ms. Tested on advanced hardware, the system proves effective in quickly tracking and identifying hazardous rockfalls, offering a significant advancement in disaster management and prevention.

## 1. Introduction

In recent years, the world has faced persistent extreme weather events, leading to significant economic losses and serious impacts on human safety. In 2015, a 7.8 magnitude earthquake in Nepal resulted in 449 deaths and widespread devastation, with nearly 90% of buildings in the worst-hit districts damaged. The economic impact amounted to losses equivalent to Nepal’s annual GDP, necessitating at least USD 5 billion for reconstruction [1]. In 2013, in Colorado, USA, a tragedy occurred when massive boulders, some exceeding 100 tons and similar in size to cars, unexpectedly tumbled from a cliff at an elevation of 4267 m on Princeton Mountain, causing casualties [2]. On 31 August 2013, heavy rainfall led to a rockfall disaster in Badouzih, Keelung [3]. The impact of rockfall on the retaining structures of a mountain road in Taiwan was assessed in a case study, revealing significant damage to the retaining structures of a mountain road (Miao 62 Line) in Miaoli County, Taiwan [4]. These incidents underscore the necessity for effective management and prevention strategies for rockfall. Further exploration on these topics can be found in studies from *Geoenvironmental Disasters* and *Social Sciences* [5,6], with innovative approaches to rockfall monitoring and early warning systems detailed in a *Scientific Reports* article [7,8,9] and strategies for mitigation, especially in Colorado’s mountainous terrain, provided by the Colorado Geological Survey [10,11].

Given that mountainous slopes constitute a significant portion, approximately three-quarters, of Taiwan’s land area [12,13,14], Taiwan faces a high occurrence rate of rockfall disasters. This has resulted in numerous casualties and an increase in national compensation cases. Moreover, rockfall disasters significantly impact transportation safety, with approximately one-third of travel time disruption in Taiwan’s eastern region being attributed to rockfall incidents. For instance, in Taroko National Park, the estimated annual loss in tourism revenue amounts to around TWD 20 billion due to frequent rockfall incidents, leading to temporary closures or visitors refraining from visiting due to safety concerns. These circumstances highlight the urgency of enhancing rockfall prevention, detection, and warning capabilities. The occurrence of these events underscores the threats posed by climate change globally and emphasizes the importance of prevention and mitigation measures against extreme weather events [1,5]. To minimize the impacts of disasters on society and the economy, governments and relevant organizations worldwide need to strengthen disaster risk management, promote climate change adaptation measures, and enhance public awareness and response capabilities. These efforts will help reduce the irreversible consequences of future disasters. Therefore, this paper proposes a rockfall hazard identification system. The system is expected to be deployed on roadside cameras. Images from cameras along roads prone to disasters will be fed into a computing system for real-time monitoring. When a disaster occurs, the system can quickly issue alerts by sending notifications through LINE messenger and email, along with photographs from the current monitoring footage to facilitate subsequent assessment. One-dimensional sensing data such as tilt and vibration will be presented on the Grafana information platform. If a suspected rockfall impact on protection nets is detected, a trigger value will be activated, prompting a red alert to notify monitoring personnel. The system could enhance rockfall disaster prevention, detection, and warning capabilities.

This rockfall hazard identification system contributes to multiple United Nations Sustainable Development Goals (SDGs). By quickly identifying and providing early warning for rockfall disasters, it helps improve the disaster resilience of road and transportation infrastructure (SDG 9), reduce risks of disruptions, and enhance overall infrastructure resilience. Rockfall disasters pose threats to the safety of urban populations and transportation systems. The application of this system can mitigate such risks, facilitating the creation of inclusive, safe, resilient, and sustainable cities and communities (SDG 11). Moreover, extreme weather conditions exacerbated by climate change often trigger rockfall and other disasters. This system enhances capabilities to respond to climate-related disasters, strengthening disaster prevention and management related to climate change impacts. This advancement positively contributes to taking urgent action to combat climate change in line with SDG 13. Therefore, this rockfall monitoring system supports the realization of key sustainable development targets centered on resilient infrastructure, sustainable cities and communities, and climate action.

In the past, due to hardware limitations, complex deep learning algorithms such as neural networks were not widely supported for object detection and tracking, leading to a reliance on traditional image processing techniques. It is important to note that rock texture patterns present similar challenges for image recognition across different environments. The intricate and irregular surface features of rocks, along with varying lighting conditions and backgrounds, make it challenging to accurately detect and identify rock formations from visual data. This complexity is consistent across various settings, highlighting the universal difficulty in processing and analyzing rock textures and forms through visual data. Methods based on polygonal Haar-like features were employed [15]. For rock detection, a Random Forest (RF) model using Support Vector Machine (SVM) and Histogram of Oriented Gradients (HOG) features was utilized [16]. Kalman filtering was applied for expanded space Multi-Criteria Evaluation (MCE) aggregation in slope sliding monitoring [17], while a combination of photogrammetry and optical flow was used for rockfall slope monitoring [18].

These techniques required manual feature design and were sensitive to factors such as lighting and background, resulting in subpar detection and tracking performance. In the past, object detection and tracking heavily relied on these traditional image processing techniques. However, with advancements in hardware and technology, modern approaches increasingly incorporate complex deep learning algorithms to achieve more accurate and efficient object detection and tracking. With the continuous advancement of hardware devices and artificial intelligence technology, we no longer rely solely on computers and central processing units (CPUs) for complex computing tasks. Specifically, Nvidia’s Cuda [19] has opened up a new era by enabling computations on graphics processing units (GPUs), further driving the development of computer vision, machine learning, and deep learning. These significant advancements have not only propelled research progress in various fields but have also laid a solid foundation for the study of rockfall recognition systems. As for specific application examples, machine learning and deep learning models have been widely applied in various domains. In a research study related to machine learning, researchers employed classification and regression methods for rockfall prediction tasks [20]. The study develops a warning system integrating various sensors with a logistic regression model to predict rockfall occurrences along a mountainous road. It classifies hazard levels into low, medium, and high based on rockfall occurrence probabilities, aiming to enhance traffic safety by providing dynamic alerts. In another research report, researchers utilized the XGBoost [21] algorithm from gradient boosting methods, specifically designed for rock classification tasks. In the field of object detection, despite the successes achieved by deep learning models such as Faster-RCNN [22], SSD [23], and RetinaNet [24], directly applying these models to tracking tasks may not always yield satisfactory results. Especially in complex and dynamic backgrounds, such as branches, leaves, and everyday structures, they can generate significant amounts of noise, posing significant challenges for object tracking and recognition tasks.

To address these issues, we have made a series of improvements and optimizations to the existing algorithms. Firstly, we combined the popular image detection model MobileNet with SSD [25]. This combination not only enables the effective tracking and detection of targets but also exhibits highly efficient characteristics. Secondly, we integrated Faster-RCNN with OpenCV’s CSRT [26] for improved tracking results. Lastly, we enhanced RetinaNet by optimizing the feature extraction layer and adjusting the size and quantity of detection boxes, further enhancing the model’s detection performance. By implementing these strategies and techniques, we can effectively improve the tracking and detection performance of the models in the presence of complex and dynamic backgrounds. This has significant academic and practical value for our research work. These studies not only demonstrate the power of these advanced computational methods but also reveal their wide applicability across various domains, including research and applications in rockfall recognition systems.

This study focuses on enhancing rockfall detection by optimizing existing algorithms and integrating deep learning models for superior tracking, along with adjustments for improved detection in complex scenes. Key objectives include surpassing target frame rates for real-time recognition, increasing accuracy in tracking the trajectories of falling objects, and pioneering the use of RGB images for rockfall recognition. These efforts contribute significantly to both academic research and practical disaster prevention applications.

## 2. Methods

In our study, we developed an innovative object tracking technique that integrates deep learning with motion analysis and data association methods. Initially, we use the Motion History Image (MHI) [27] algorithm to capture motion trajectories of objects in videos. Next, we apply YOLO, a deep learning-based object detection system, to identify targets and evaluate their confidence levels. Our model, trained on the COCO dataset and further refined with our unique dataset of falling rocks, undergoes 300 training cycles, optimizing for minimum loss. Utilizing the Nvidia 4090 GPU, we enhance the processing speed for image analysis, overlaying the results with MHI for accurate detection. Target positions in successive frames are estimated using the Kalman filter and the Hungarian algorithm, with the Euclidean distance aiding in ID assignment [28]. Finally, the Perceptual Hash Algorithm and template matching techniques are employed to boost tracking and detection reliability.

### 2.1. Optimization of Motion History: Enhancing Image Change Effectiveness through Internal Parameter Optimization

In the process of image analysis, we first convert the images to grayscale, which effectively reduces computational complexity and accelerates subsequent processing. When analyzing motion history trajectories, we select four frames as a processing unit and perform image differencing analysis. Specifically, we add the first *F*^1*st*^ and second frames *F*^2*nd*^ to obtain preliminary image changes $F_{1}^{Sum}$. Then, we overlay the third frame *F*^3*rd*^ with the previous two frames in an 8:2 ratio to obtain $F_{2}^{Sum}$. Subsequently, we perform differencing between this overlaid image $F_{2}^{Sum}$ and the fourth frame *F*^4*th*^ to obtain further image changes. *α*, *β*, *γ*, and *ω* represent the i. The equation is as follows:

(1) $$F_{1}^{Sum}={\alpha \times F}^{1st}+{\beta \times F}^{2nd}$$

(2) $$F_{2}^{Sum}=\gamma \times F_{1}^{Sum}+\omega {\;\times \;F}^{3rd}$$

(3) $$\delta^{FD}=\left|\left(F_{2}^{Sum}-F^{4th}\right)\right|\;$$

The next step involves the binarization of the images, which helps us to differentiate and label the regions of interest more clearly. In grayscale images, 0 represents full black, while 255 represents full white. We choose a threshold of 32 for binarization, meaning that we label regions with grayscale values greater than 32 as 255 (full white) and regions with grayscale values less than or equal to 32 as 0 (full black). The threshold value of 32 is experimentally determined and effectively distinguishes motion trajectories from the background. $I_{Initial}^{gray}$ is the initial gray image and $I_{Binary}^{gray}$ is the image after the binary preprocess. The equation is as follows:

(4) $$I_{Binary}^{gray}=\left\{\begin{array}{c} 255,\;if\;I_{Initial}^{gray}>32\\ 0,\;if\;I_{Initial}^{gray}\leq 32 \end{array}\right.\;$$

**Figure 1:**
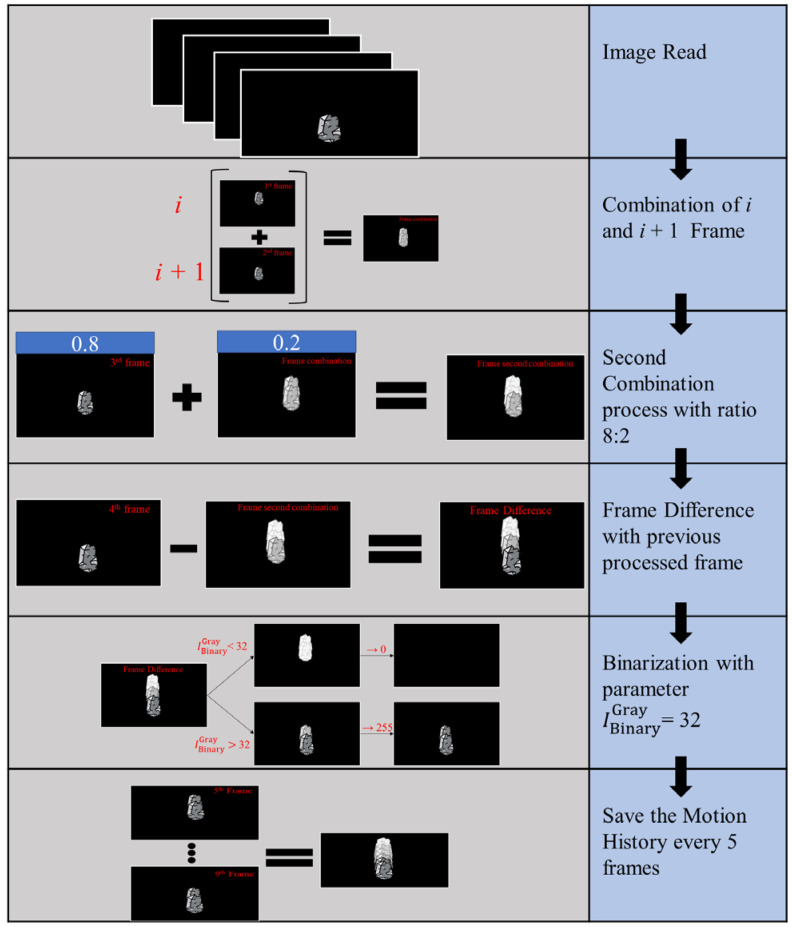
This figure summarizes the entire optimized motion history analysis process, including image overlay, image differencing, binary thresholding, and the acquisition of motion trajectories from short-term motion variations to long-term historical tracks.

After obtaining the binarized image, to better observe and analyze the motion trajectories, we choose to retain the history of the previous five frames. This step helps us to better understand the motion patterns and regularities of moving objects. Overall, this grayscale-based method effectively reduces computational complexity and accurately extracts the desired motion trajectory information, enabling the effective identification and tracking of moving objects. The entire processing workflow is illustrated in Figure 1.

### 2.2. YOLO

YOLOv5 is an advanced deep learning model for object detection and recognition. It introduces improvements such as the Focus module and CSP structures to enhance detection accuracy and efficiency. It leverages FPN and PAN for feature fusion, improving the model’s ability to handle objects of different sizes and proportions. Additionally, it employs data augmentation techniques to increase the diversity and variability of the training data, improving the model’s generalization and robustness. YOLOv5 is widely used in various applications and domains.

### 2.3. Kalman Filter

Kalman filtering is a dynamic model for systems, such as the physical laws of motion, that estimates the system’s change based on known sequential measurements. It is a common sensor and data fusion algorithm. In this study, Kalman filtering is used to predict the coordinates of falling rocks in the image. There are two main methods in the Kalman filtering process: Predict and Correct. Below are the formulas and explanations for each parameter in each method:

(1) Prediction Step (Predict):

In this stage, we first update the predicted value *u* using the state transition matrix *F*. This update process can be achieved by multiplying the state transition matrix *F* with the current predicted value *u*, as shown in Formula (5). Next, we need to update the predicted error covariance matrix *P.* The calculation involves multiplying the state transition matrix *F* with the current predicted error covariance matrix *P* and adding the model noise covariance matrix *Q*, as shown in Formula (6).

(5) u′=F∗u

(6) $$P\prime =F*P*F^{T}+Q$$

(2) Correction Step (Correct):

In this stage, we first compute the relevant values for the observation noise covariance matrix *R* and the observation matrix *A*, as shown in Formula (7). Then, we calculate the Kalman gain *K* and use it to correct the predicted value *u*. This correction process is based on the error between the observed value b and the predicted value, as shown in Formulas (8) and (9). Finally, we update the predicted error covariance matrix *P* based on the Kalman gain *K* and other relevant values, as shown in Formula (10).

(7) $$C=A*P\prime *A^{T}+R$$

(8) $$K=P^{\prime }*A^{T}+C^{-1}$$

(9) $$u=u^{\prime }*K*(b-A*u^{\prime })$$

(10) $$P=P^{\prime }-K*C*K^{T}$$

### 2.4. Hungarian Algorithm

We utilize the Hungarian algorithm to calculate the Euclidean distance between trajectories and detections. The Hungarian algorithm is a method for solving the assignment problem optimally. In our research, it is employed to assign the trajectories of falling rocks to the detected targets, determining which detections correspond to which tracking targets. The calculation process involves three steps as follows:

(1) Compute the cost matrix:

Given *N* trajectories and *M* detections, we first compute a cost matrix *C* of size *N* × *M*. Each element $C_{ij}$ represents the Euclidean distance between the predicted position of trajectory *i* and detection *j*, as shown in Formula (11).

(11) $$C_{ij}=\sqrt{{\left(x_{i}-x_{j}\right)}^{2}+{\left(y_{i}-y_{j}\right)}^{2}}$$

(2) Assign trajectories and detections using the Hungarian algorithm:

We create a matrix *X* of size *N* × *M*. When trajectory *i* is assigned to detection *j*, *X*[*i*, *j*] is set to 1; otherwise, *X*[*i*, *j*] is set to 0. The objective function is formulated as shown in Formula (12). Each trajectory can only be assigned to one detection, and each detection can only be assigned to one trajectory. The constraint is represented by Formula (13).

(12) $$Minf(x)=\sum_{1}^{N}\sum_{1}^{M}C\left[i,j\right]*X[i,j]$$

(13) $$\forall_{i}\;\in \;\left\{1,2,\ldots ,N\right\},\;\sum_{j=1}^{M}X\left[i,j\right]=1,\;\;\forall_{j}\;\in \;\left\{1,2,\ldots ,M\right\},\;\sum_{i=1}^{N}X\left[i,j\right]=1$$

(3) Update the trajectory states:

For each trajectory assigned to a detection, we update its state using the Kalman filter. If a trajectory is not assigned to any detection, its state prediction remains unchanged.

### 2.5. Normalized Cross-Correlation

In the process of normalized cross-correlation [29] matching, we compare the similarity between a template image (template) and a region of the target detection image of the same size. By calculating the correlation between the template and the image region, we can identify the region that best matches the template. The calculation process is shown in Formulas (14) to (18):

(1) Compute the pixel intensity difference between the template *T* and the image region *I*:

(14) $$\Delta I_{ij}=T\left(i,j\right)-mean\left(T\right),\;\Delta T_{ij}=I(x+i,y+j)-mean\left(I\left(x,y\right)\right)$$

(2) Compute the product of the differences:

(15) $$\prod =\Delta I_{ij}*\Delta T_{ij}$$

(3) Sum all the products:

(16) $$Sum=\sum_{i=1}^{N}\sum_{j=1}^{M}\Delta I_{ij}*\Delta T_{ij}$$

(4) Calculate the product of the standard deviation of the template *T* and the image region *I*:

(17) $$\sigma =\sigma_{T}*\sigma_{I}(x,y)$$

(5) Combine the sum of the products with the product of standard deviation and multiply by a constant:

(18) $$R\left(x,y\right)=\left(\frac{1}{N-1}\right)*\left(\frac{Sum}{\sigma }\right)$$

### 2.6. Difference Hash Table

Differential hashing [30] is a method for computing image hash values that enables the fast comparison of the similarity between two images. It compresses the images to a specific aspect ratio, such as a 9 × 8 pixels matrix, calculates the grayscale intensity value for each pixel, converts it to binary, and then computes the binary difference between adjacent elements. Finally, it concatenates all the binary values to obtain the image hash. The process can be summarized in three steps as follows:

(1) Resize image and convert to grayscale:

The image is resized to a specific size, such as 9 × 8, and then converted to grayscale intensity values ranging from 0 to 255.

(2) Compute differences:

Calculate the grayscale intensity difference between adjacent pixels. If the intensity of the first pixel is greater than the second pixel, it is assigned a value of 1; otherwise, it is assigned a value of 0. This is represented by Formula (19).

(19) $$D(x)=\left\{\begin{array}{c} 1,\;if\;p_{i,j}>p_{i,j+1}\\ 0,\;if\;p_{i,j}<p_{i,j+1} \end{array}\right.$$

(3) Compute the hash value:

First, convert the binary differences to decimal values to obtain the decimal hash value $h_{i}$ for each row. Then, concatenate all the row hash values to generate the differential hash value Dhash, as shown in Formula (20).

(20) $$h_{i}=\sum_{j=1}^{N}2^{j-1}*D(x)\;Dhash=\left[h_{1},\cdots ,h_{N}\right]$$

### 2.7. Overview the System

**Figure 2:**
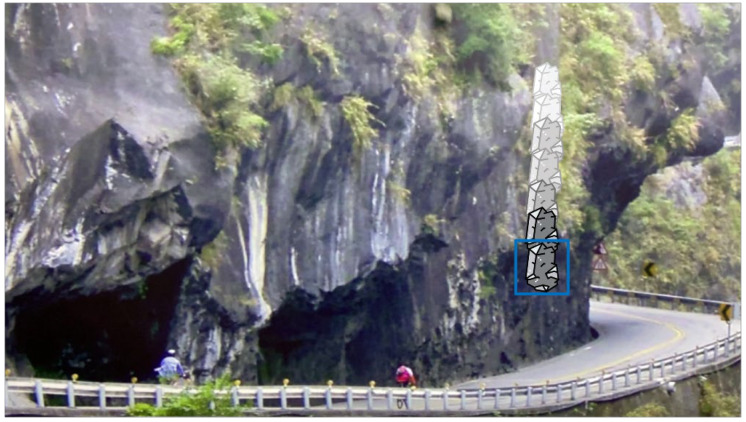
Blue rectangular boxes indicating the trajectory of the falling object.

**Figure 3:**
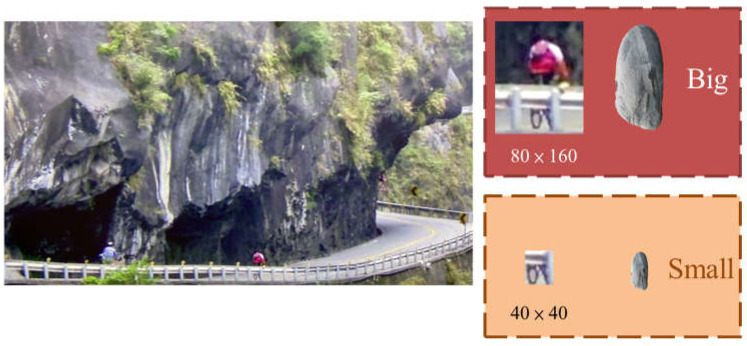
The size definition on the image in pixel values.

Before starting the analysis, some basic processing and calibration are applied to the collected images. The input resolution of the images is 1920 × 1080, with a frame rate of 30 FPS, and they are captured in RGB color mode. The images are obtained from two lenses with different focal lengths (26 mm and 52 mm), each with a resolution of 12 million pixels. The process can be divided into two parts. Firstly, the image input undergoes image differencing and motion history trajectory calculation, and the result is output as the red channel. The foreground is considered as moving objects, while the background is considered as non-moving objects. The trajectory of the target is marked with a blue rectangular box, as shown in Figure 2. For the size of rockfalls that cause serious damage, we define 80 × 160 as big rockfall and 40 × 40 as small rockfall, shown in Figure 3. Secondly, YOLO is used for image prediction, and the predicted ROI objects are extracted and output as the green channel along with their confidence scores. These confidence scores are saved in the image information.

Next, the red channel of the MHI and the green channel of YOLO are overlaid to create the overlaid image. In this overlaid image, the yellow area represents the overlap between the two channels, and we can process this yellow overlap region. Then, we obtain the coordinates of the yellow area and use the Kalman filter to predict their next position. Then, we assign an ID to each target using the Hungarian algorithm. This approach allows us to effectively detect multiple targets even when they appear simultaneously, and the results are saved in the image information.

**Figure 4:**
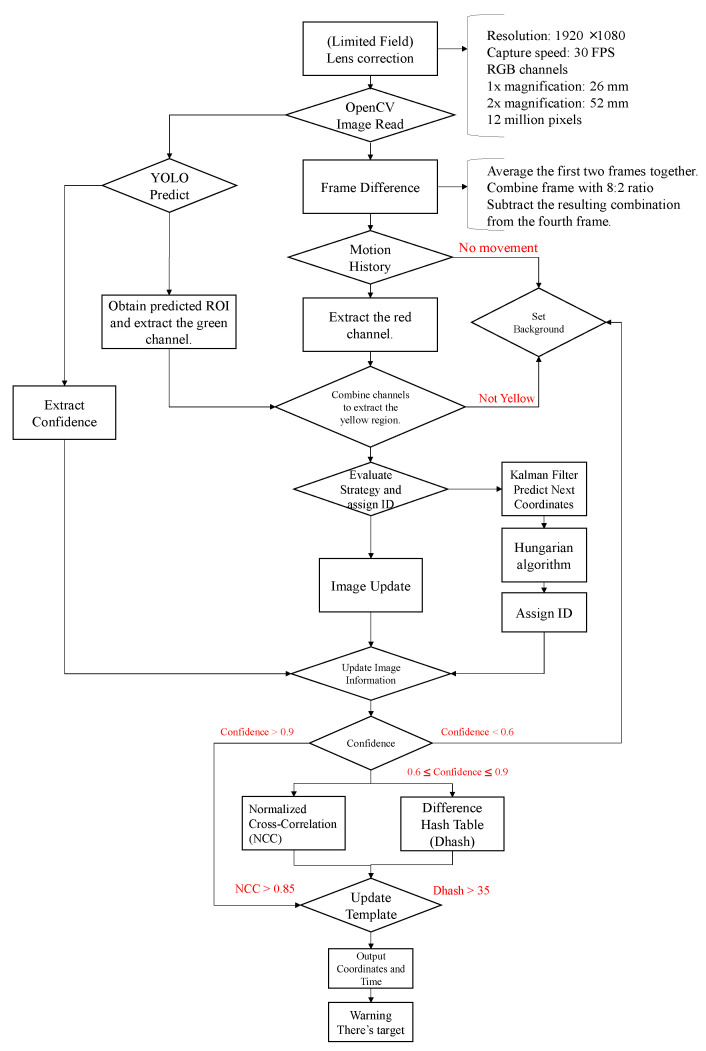
This flowchart illustrates the process starting from the input of the initial image, which undergoes preprocessing using the OpenCV module. It then proceeds to target detection through the YOLO deep learning model, followed by data processing involving coordinate tracking and template matching on the images. This process aims to achieve the final tracking and detection of rockfall, ultimately outputting warnings based on the detection of rockfall.

Finally, a decision strategy is applied to interpret the image information. When the predicted confidence score is greater than 0.9, the target is considered as a foreground falling object. When the predicted confidence score is lower than 0.6, the target is considered as background. When the predicted confidence score is between 0.6 and 0.9, two algorithms are used: normalized correlation matching and the difference hash table. The difference hash table is compressed into a 9 × 8 matrix. If the normalized correlation matching value is greater than 0.85 and the hash value of the difference hash table is greater than 35, we update our template and output the coordinates of the target and the current time, generating a warning in the terminal about the possibility of falling rocks. This process demonstrates our image processing and analysis methods, utilizing mathematics and computer vision techniques for tasks such as image calibration, target detection, trajectory prediction, and target identification, and integrating the results into a single workflow, as shown in Figure 4.

## 3. Datasets

In our research, we conducted a simulated experiment in a small-scale field to simulate the occurrence of falling rocks in real-life situations. By conducting such a localized and on-site simulated experiment, we can gain a deeper understanding of real-world falling rock scenarios and optimize our system to better adapt to these situations. Such on-site experiments provide a practical and intuitive evaluation criterion to help assess and improve our methods and techniques.

**Figure 5:**
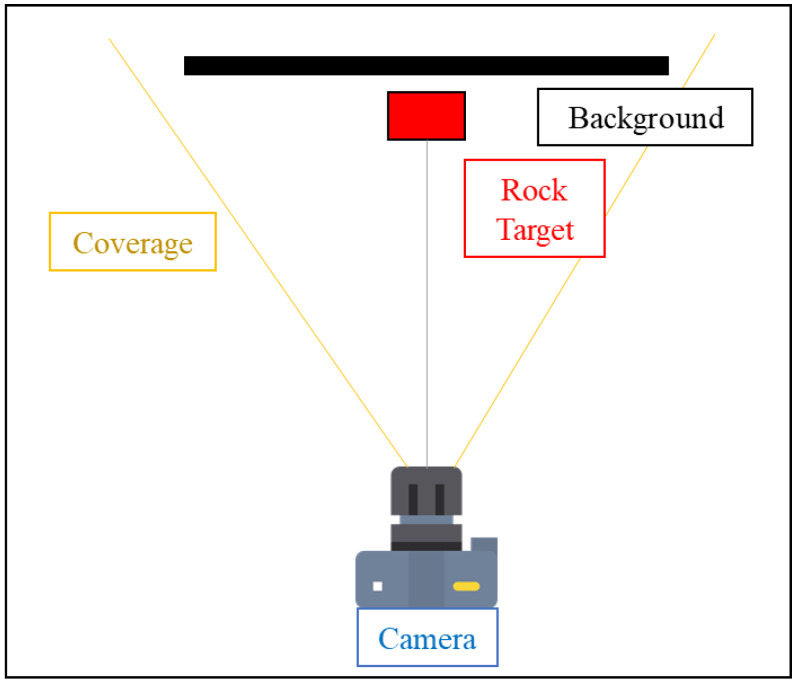
The experimental setup diagram. The structure is divided into three components: the camera, the rock target, and the background.

**Figure 6:**
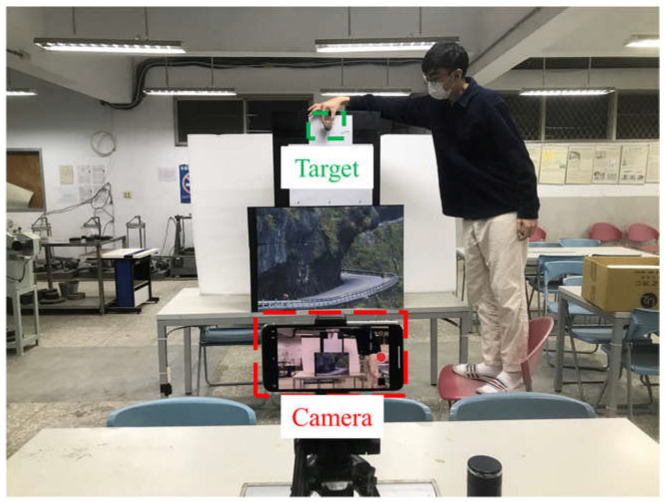
In the actual experiment, one person will release stones in compliance with the field conditions to simulate high-speed free-falling stones dropping from outside the scene into the scene.

The optical measurement device is the iPhone 13 Pro, which has an aperture of f/2.8, and we used two different focal lengths, 26 mm and 52 mm. To ensure the accuracy of the test results, we maintained a distance of 2 m between the camera and the target object, as shown in Figure 5 and Figure 6.

In our research, we conducted shooting and testing from various angles and backgrounds, including scenarios where both people and rocks coexist. These shooting and testing results were organized into a dataset that we used for pre-training YOLO to obtain training weights for rock detection. To improve the accuracy of the training results, we employed a data augmentation strategy.

Our training set consists of self-captured and self-annotated 1920 × 1080 RGB images. The annotation principle we followed was to outline the edges of the target objects with bounding boxes. To ensure our model can respond appropriately to various scenarios, our dataset covers different situations such as single and multiple targets and moving and stationary rocks. In the end, our dataset consisted of 2298 training images and 192 validation images. We applied nine different image augmentation techniques, and the details of these techniques are as follows: (1) Flip: Ho Flip: Horizontal; (2) 90° Rotate: Clockwise, Counter-Clockwise; (3) Rotate: Between −15°and +15°; (4) Shear: ±15° Horizontal and ±15° Vertical; (5) Saturation: Between −25% and +25%; (6) Brightness: Between −25% and +25%; (7) Exposure: Between −25% and +25%; (8) Blur: Up to 10px; and (9) Noise: Up to 5% of pixels. The choice of parameters and settings, particularly in data augmentation, is driven by the need to enhance the model’s generalization ability by exposing it to a wide variety of data scenarios.

## 4. Results

In our research experiment, the model was trained for 300 epochs. During this process, we set several parameters: an initial learning rate of 0.01, the first-order momentum parameter (beta1) of the Adam optimizer to 0.937, a weight decay parameter of 0.0005, and the number of anchors per output layer set to three. We also set the training threshold for Intersection over Union (IoU) to 0.2. These settings aimed to optimize the learning efficiency of our model and reduce the risk of overfitting, resulting in the stable convergence of the training process.

### 4.1. Training Results

**Figure 7:**
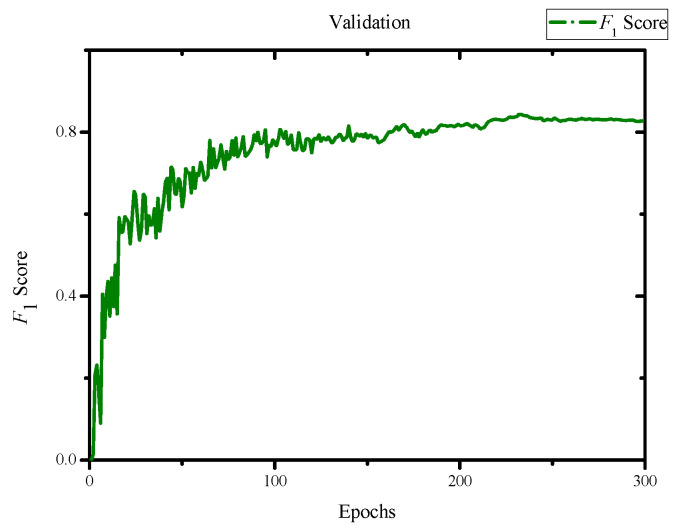
Results of *F*_1_ score with scores of 0.84.

**Figure 8:**
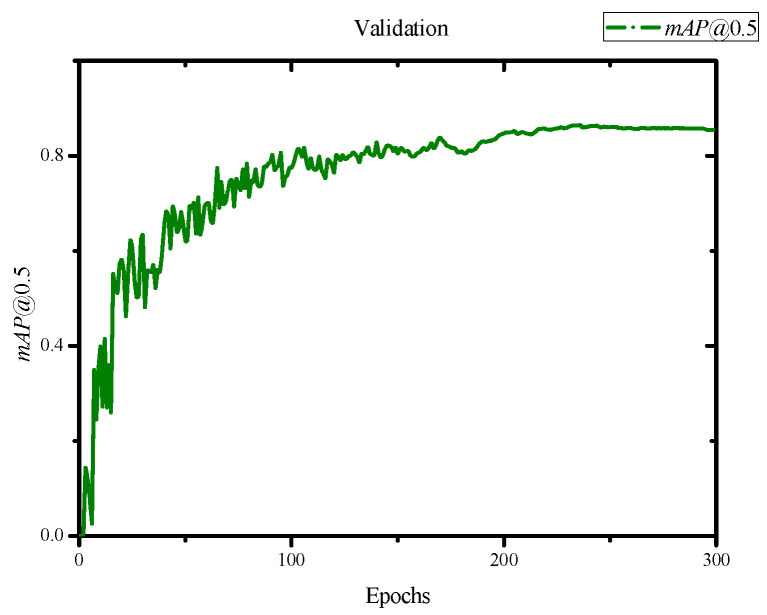
Results of *mAP* at 0.5 with scores of 0.845.

**Figure 9:**
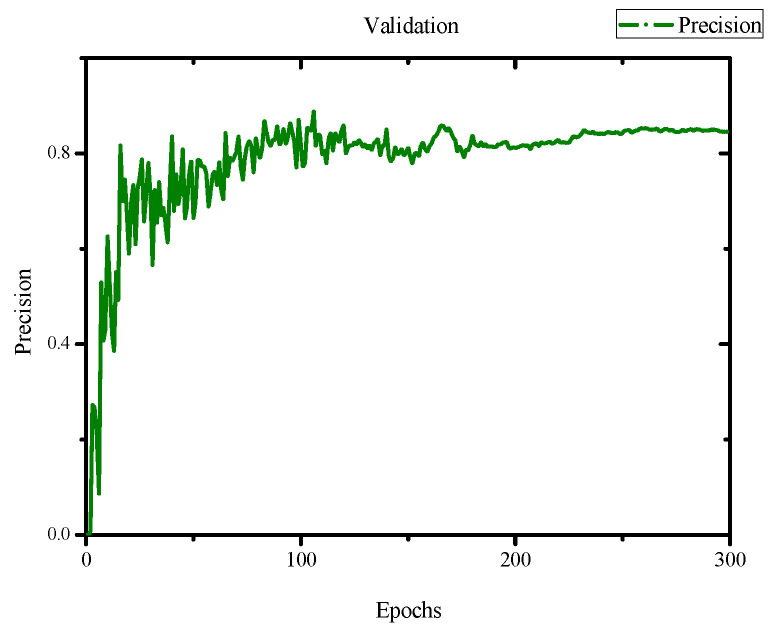
Results of precision with values of 0.93.

**Figure 10:**
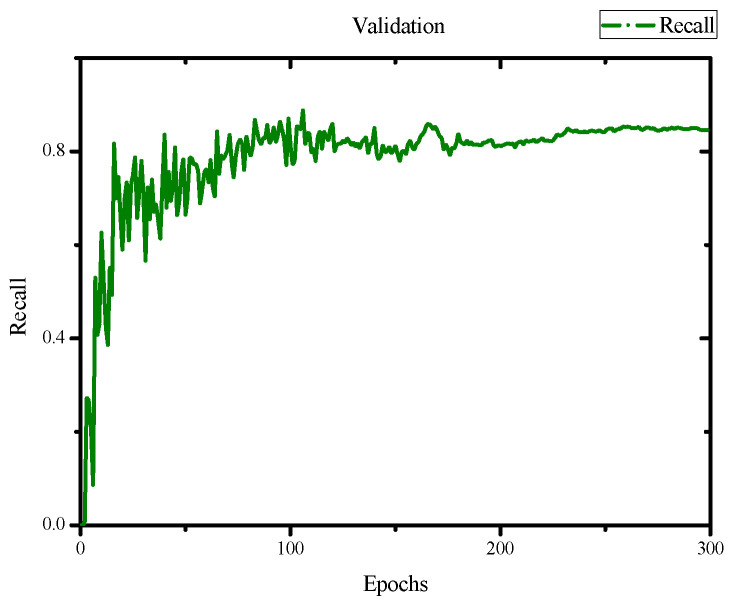
Results of recall with values of 0.91.

**Figure 11:**
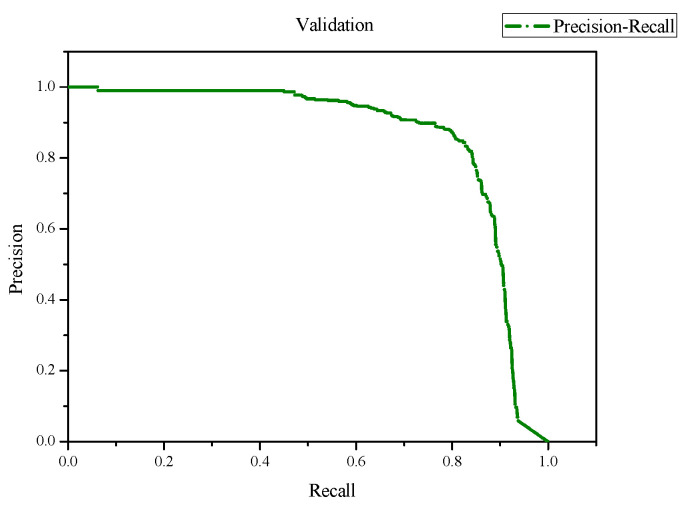
Results of the Precision–Recall figure.

In the evaluation of the training results, we obtained an F1 score of 0.84 and a mean Average Precision (mAP) score of 0.845 with an IoU threshold of 0.5, as shown in Figure 7 and Figure 8. Additionally, we achieved a precision of 0.82 and a recall of 0.81, as depicted in Figure 9 and Figure 10. Lastly, the Precision–Recall is shown in Figure 11. Overall, these evaluation results demonstrate that our model has achieved good learning effectiveness and performance during the training process.

### 4.2. Prediction Results

In our experimental research, we used an Nvidia RTX 3060 laptop graphics card and an i7-12650H processor for computational processing. The shooting parameters were based on our device and experiment. We conducted tests in two main scenarios: single-object detection and multi-object detection. For each scenario, we performed image capture tests with both 1× and 2× focal length settings. The images captured with the 2× focal length setting were not annotated. All test videos were new data that did not appear in the training or validation datasets, ensuring that our test results reflect the model’s generalization capability. The relevant parameters and settings for these test videos are detailed in Table 1. The results are shown in Table 2.

(1) Single Object; 1×: When detecting large-sized falling rocks that are comparable in size to a human, the target object is effectively captured in every frame of its falling process. Each frame can be successfully captured.
(2) Single Object; 2×: When detecting falling rocks with a 2× focal length, detection is only possible in simple background conditions and in very few cases. In complex backgrounds, detection is not possible at all. Our training dataset did not include annotations for images captured with a 2× focal length.
(3) Multi-Object; 1×: When detecting multiple targets, the results are similar to the single-object scenario, where falling rocks can be detected even in complex backgrounds. However, due to limitations in the test environment, there may be some shadows that affect the detection performance.

### 4.3. Field Prediction Results

To enhance the dataset’s diversity, we plan to incorporate a broader selection of videos that encompass a variety of zoom levels. This expansion will enable the model to more effectively learn and recognize landslide characteristics under diverse observational conditions, thereby enhancing its generalization capabilities. The attached videos https://youtu.be/Xq9FlsVN-A8 (accessed on 24 March 2024) and https://youtu.be/nEnuFX-QBJk (accessed on 24 March 2024) showcase the results of field tests, demonstrating the utility of our approach in real-world applications.

## 5. Discussion

Existing solutions are based on sensors deployed on rockfall protection nets, and data analysis is performed on these sensors. However, these approaches are geared towards analyzing data from rockfall disasters that have already occurred. Our solution focuses on real-time detection in specific road sections.

In our experiment, considering the scenario of the roadside camera, we adopted a single lens for shooting. Therefore, we did not perform conversions for image and distance, relying solely on pixels.

**Figure 12:**
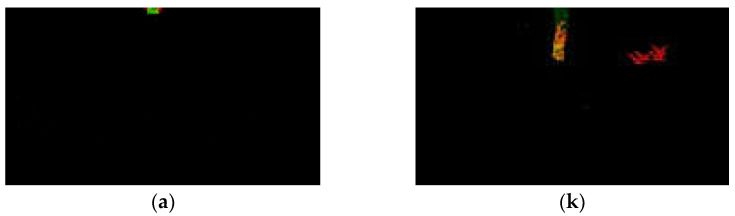

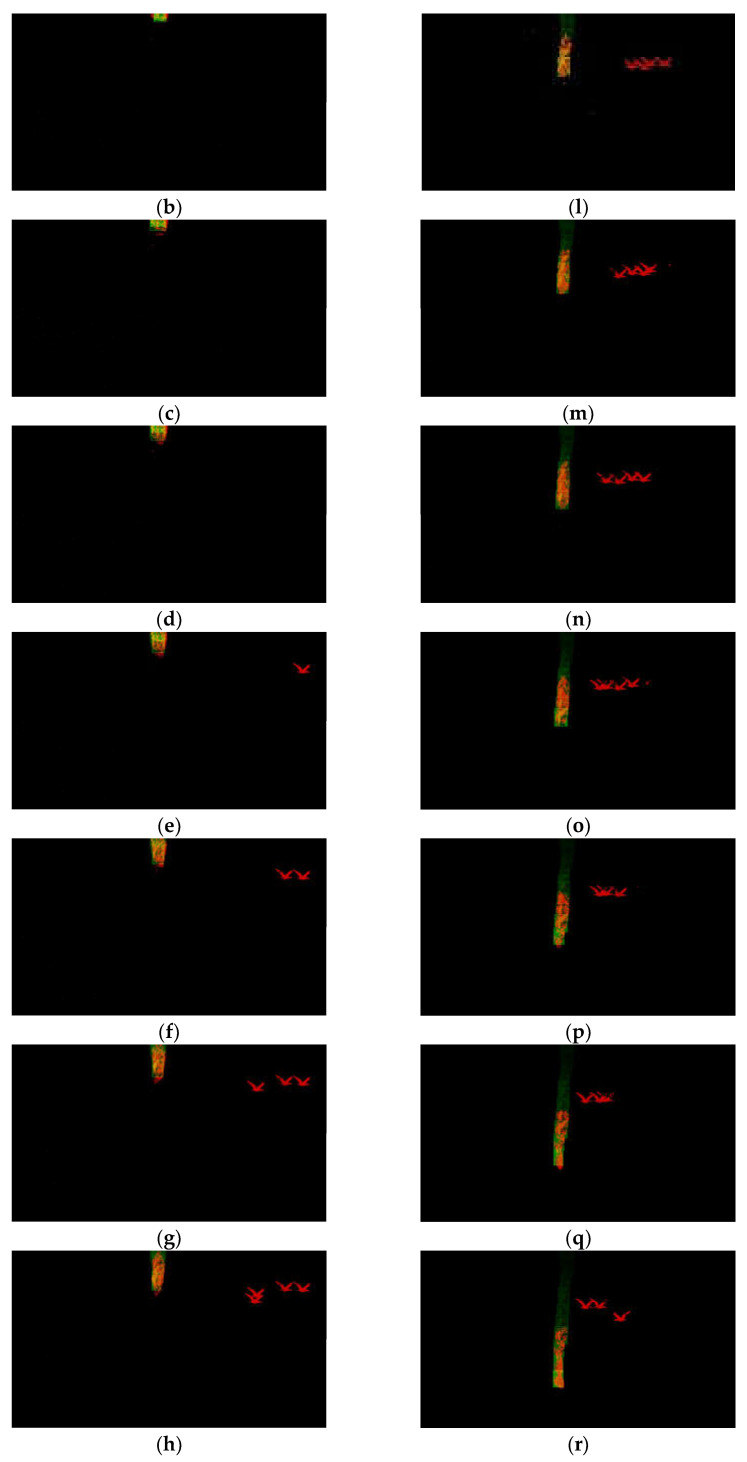

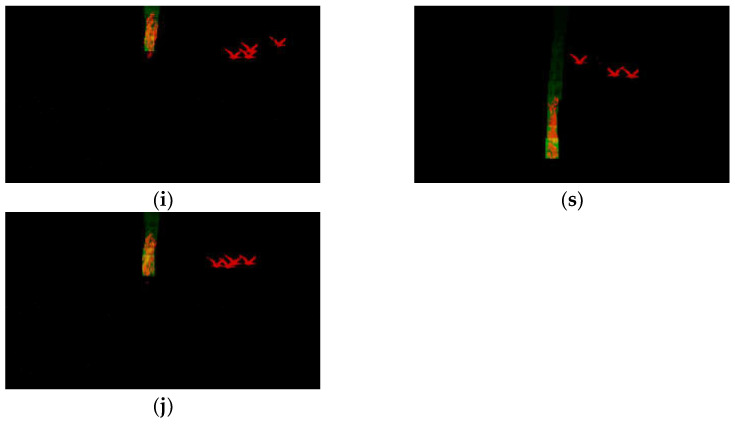
The complete and well-defined trajectory of a falling rock from (**a**–**s**) corresponds to frames 82 to 100 in the experimental sequence, which is the main focus of the experiment.

However, among all the rockfall detection systems, it is rare to use image recognition and tracking for identifying and tracing falling rocks. Comparing our system with others is challenging. And it is also rare to capture birds and falling rocks in the wilderness; we conducted experiments using manually labeled bird species and rocks in a small experimental field with complex backgrounds. The experiments indicated that we can accurately distinguish between two common objects in the mountains, falling rocks and birds, as shown in Figure 12. In the scenario of rock falling, our system will calculate information locally in real-time. The local system is expected to be installed on-site and transmit short messages via wireless networks to notify nearby pedestrians. The combination of short text messages and real-time calculations enables the system to achieve the real-time notification of falling rocks for pedestrians.

In our dataset testing, we discovered that the detection of small rockfall targets was not ideal. Although we chose not to apply blurring filters to the images in order to maintain their authenticity, we found that preprocessing with bilateral filtering to remove noise can significantly improve image recognition accuracy and reduce noise during YOLO detection. Compared to the Distributed Acoustic Sensing (DAS) approach [8], which utilizes fiber-optic technology for detailed analysis and monitoring, our proposed system provides a forward-looking solution for real-time detection and disaster prevention. It offers additional advantages, such as the capability to monitor actual rockfall locations and assess the volume of rockfall spaces.

## 6. Conclusions

In this study, we have designed and implemented a rockfall detection and tracking system tailored for cliffs. The system adeptly navigates the detection challenges posed by complex backgrounds. Through the analysis and processing of a dataset comprising 2490 RGB rockfall images and 30 high-definition (1920 × 1080 resolution) rockfall test videos, our algorithm exhibits strong performance in detecting large-sized rockfalls. Despite not testing different zoom levels in the training dataset, our system showcases remarkable generalization capabilities. Moreover, with an execution time of 12.9 FPS on a GPU, our rockfall recognition system achieves rapid and efficient detection, given adequate data support. However, in tests focusing on smaller targets, we identified limitations due to the narrow scope of the annotated dataset and the suboptimal image acquisition speed, leading to potential deformations and transparency issues in the images. To mitigate these issues, we plan to broaden the dataset and enhance the image acquisition speed, aiming to improve detection performance. Additionally, we aim to relocate our experimental setup to real-world sites, like the Nine Turns Trail in Taiwan, to further refine and validate our system. For implementation, a robust computing server, wireless transmission towers, and cameras are necessary. This infrastructure ensures timely alerts for pedestrians and safety personnel, facilitating emergency road closures during rockfall incidents. In the case of an accident, instant visual data provision and streamlined post-disaster repairs are enabled by the recorded image data.

Moving forward, we intend to utilize tools like Blender to simulate real-world scenes in a virtual environment, thereby expanding our dataset with these simulations. We will assess the discrepancies between Blender-generated images and actual photos, striving to transpose the characteristics learned from real images to the Blender-generated ones using diffusion models, thus enhancing the training dataset’s quality and diversity. The Stable Diffusion Model will be employed to sketch a black mask along the predicted rockfall trajectory and generate rockfall images. However, to address the temporal sequence challenges in the rockfall trajectory, we will integrate an attention model to bolster the connections between sequential falling rockfall images, facilitating dataset generation. Moreover, we plan to manually establish a small experimental field for collecting diverse scale rockfall image data. Our research, merging image recognition and deep learning, addresses rockfall disasters with significant implications for future innovations in this domain, applicable across intelligent disaster prevention and traffic safety. Our real-time image recognition and detection system marks a leap towards identifying dynamic objects in scenarios such as debris flows and avalanches, contributing to the evolution of intelligent transportation and vehicle safety systems.

## Figures and Tables

**Table 1:** Testing video table.

Task	Speed (FPS)	Target	Focal Length	Testing Videos
System	30	Single	26	14
	30	Single	52	7
	30	Multiple	26	6
	30	Multiple	52	3
	60	Single	52	24

**Table 2:** Testing results.

Task	Speed (FPS)	Target	Rockfall Size	Focal Length	Undetected Percentage
System	30	Single	Big	26	16/95
	30	Single	Small	26	62/94
	30	Single	Big	52	18/37
	30	Single	Small	52	18/24
	30	Multiple	Big and small	26	45/87
	30	Multiple	Big and small	52	20/28
	60	Single	Big	52	12/144
	60	Single	Small	52	46/151

## Data Availability

Not applicable.
